# ﻿Two new species of the genus *Samarangopus* and the first record of *Eurypauropusjaponicus* (Arthropoda, Myriapoda, Pauropoda, Eurypauropodidae) from China

**DOI:** 10.3897/zookeys.1165.102936

**Published:** 2023-06-01

**Authors:** Yan Gao, Yun Bu

**Affiliations:** 1 Shanghai Natural History Museum, Shanghai Science & Technology Museum, 200041 Shanghai, China Shanghai Natural History Museum, Shanghai Science & Technology Museum Shanghai China

**Keywords:** Hunan, pauropod, protuberances, setae, taxonomy, Zhejiang

## Abstract

Two new species, *Samarangopustestudineus***sp. nov.** from Hunan, South China and *S.rotundifolius***sp. nov.** from Zhejiang, East China, are described and illustrated. *Samarangopustestudineus***sp. nov.** is characterized by unusual testudinal patterns on the dorsal side of the body and well-differentiated marginal protuberances on tergites. *Samarangopusrotundifolius***sp. nov.** features large, round, leaf-shaped marginal protuberances and small, candle-like dorsal protuberances on tergites. Both of these species are compared to similar species in detail. In addition, *Eurypauropusjaponicus* Hagino & Scheller, 1985 is newly recorded from China.

## ﻿Introduction

Pauropoda are tiny arthropods living in litter or soil. They are among the least-known myriapods in the world, with only about 990 species described worldwide (https://www.itis.gov/, accessed May 2023). The Chinese pauropod fauna is poorly known, with only 50 species reported so far (Bu 2020a, 2020b, 2020c, 2021; Qian et al. 2018). In recent years, several interesting species of pauropods have been discovered in China: *Psammopauropusmacrospinus* Bu, 2020, family Hansenauropodidae Remy, 1954, was found in sand on the seashore and is characterized by the presence of a pair of large spines on the pygidial tergum (Bu 2020a); *Colinauropuschinensis* Bu, 2020, *C.chongzhoui* Bu, 2020, and *C.foliosus* Bu, 2020, family Colinauropodidae Scheller, 1985 can be recognized by having their tergites divided into sclerotized coarse plates (Bu 2020b), and *Dasongiusliupanensis*Bu 2021 and *D.spatulatus*, family Pauropodidae Lubbock, 1867 both have a specialized engraved, honeycomb-like surface on the pygidial tergum, which is diagnostic of the endemic genus *Dasongius* (Bu 2021).

The family Eurypauropodidae Ryder, 1879 currently includes about 70 valid species (https://www.itis.gov/). It is characterized by a flattened body, strongly sclerotized tergites with a coarsely ornamented surface, modified setae, and specialized marginal protuberances (Scheller 2011). It was first reported in China with a preliminary description of one undetermined species *Eurypauropus* sp. from Zhejiang Province (Zhang and Chen 1988). After a long gap without new discoveries in China, three species of the genus *Samarangopus* Verhoeff, 1934 have been reported: *S.dilatare* Qian, 2014 from Jiangxi Province (Qian et al. 2014), and *S.zhongi* Bu, 2020 and *S.canalis* Scheller, 2009 from Tibet (Bu 2020c). During an extensive soil-fauna investigation in eastern and southern China from 2012 to 2020, some specimens of the family Eurypauropodidae were obtained. Two of them are new species of the genus *Samarangopus* and one belongs to the genus *Eurypauropus*, which is recorded here for the first time in China. Here, *S.testudineus* sp. nov. and *S.rotundifolius* sp. nov. are described and illustrated, and *Eurypauropusjaponicus* Hagino & Scheller, 1985 is described and illustrated based on Chinese specimen.

## ﻿Materials and methods

All pauropods were obtained by extraction of soil and litter samples from broad-leaf or mixed forests using Berlese–Tullgren funnels. Specimens were sorted under a stereomicroscope and preserved in 80% alcohol. They were mounted on slides using Hoyer’s solution and dried in an oven at 50 °C. Observations were performed under a phase contrast microscope (Leica DM 2500). Photos were taken using a digital camera (Leica DMC 4500). Line drawings were made using a drawing tube. All specimens are deposited in the collection maintained by the Shanghai Natural History Museum (**SNHM**), Shanghai, China.

Abbreviations used in the descriptions follow Qian et al. (2018). Absolute lengths of all body parts are given in mm and μm. Remaining measures in the text refer to relative lengths. In the description of the new species, measurements and indices of paratypes are given in brackets.

## ﻿Results

### ﻿Taxonomy

**Family Eurypauropodidae Ryder, 1879**

#### ﻿Genus Samarangopus

Taxon classificationAnimaliaTetramerocerataEurypauropodidae

Verhoeff, 1934

AD2D9E9C-7D87-5725-A829-73F138D369BD

##### Type species.

*Samarangopusjacobsoni* (Silvestri, 1930).

#### ﻿ Samarangopus testudineus sp. nov.

Taxon classificationAnimaliaTetramerocerataEurypauropodidae

F695BFC6-7838-5A5A-B5CD-88CC75C5EFE9

https://zoobank.org/5E05034A-D037-4779-AEA9-044FFA22CEFF

Figs 1 , 2 , 3 , 4

##### Material examined.

***Holotype***, female adult with 9 pairs of legs (slide no. HN-SHS-PA2020035) (**SNHM**), China, Hunan Province, Shaoyang City, Xinning County, Shunhuangshan Nature Reserve, extracted from soil samples in mixed forest, elev. 900 m, 26°23'N, 111°00'E, 4-IX-2020, coll. C.W. Huang. ***Paratype***, 1 juvenile with 8 pairs of legs (slide no. HN-NS-PA2020036), Hunan Province, Shaoyang City, Chengbu County, Nanshan National Park, extracted from soil samples in mixed forest, elev. 1200 m, 26°18'N, 110°29'E, 8-IX-2020, coll. C.W. Huang.

##### Diagnosis.

*Samarangopustestudineus* sp. nov. is characterized by testudinal pattern (tortoise shell-like) on the dorsal side of the body, marginal protuberances on tergites well-differentiated into four kinds of shapes, and one pair of sausage-shaped bladders on the anal plate.

##### Description.

**Figure 1.**
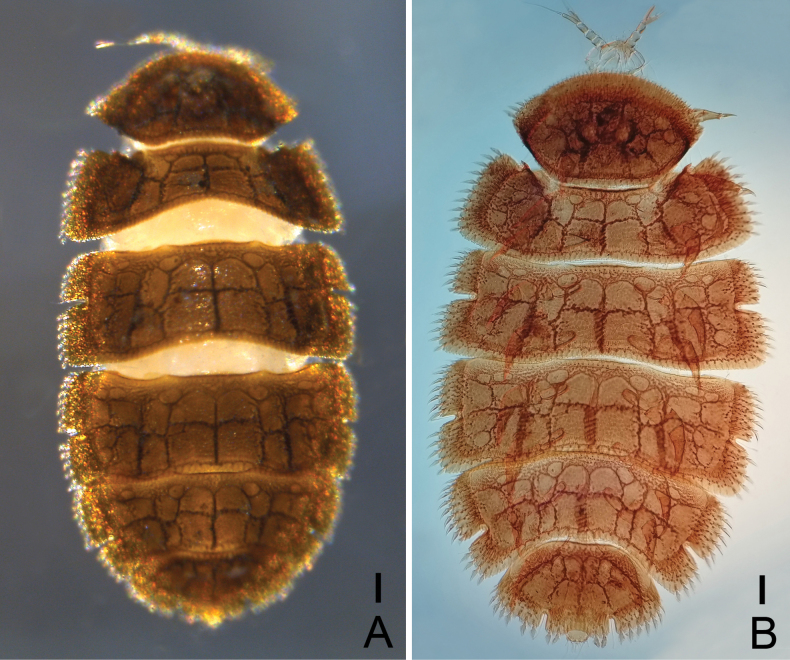
*Samarangopustestudineus* sp. nov. (holotype) **A** habitus, dorsal view, in alcohol **B** habitus, dorsal view, on slide. Scale bars: 100 μm.

**Figure 2.**
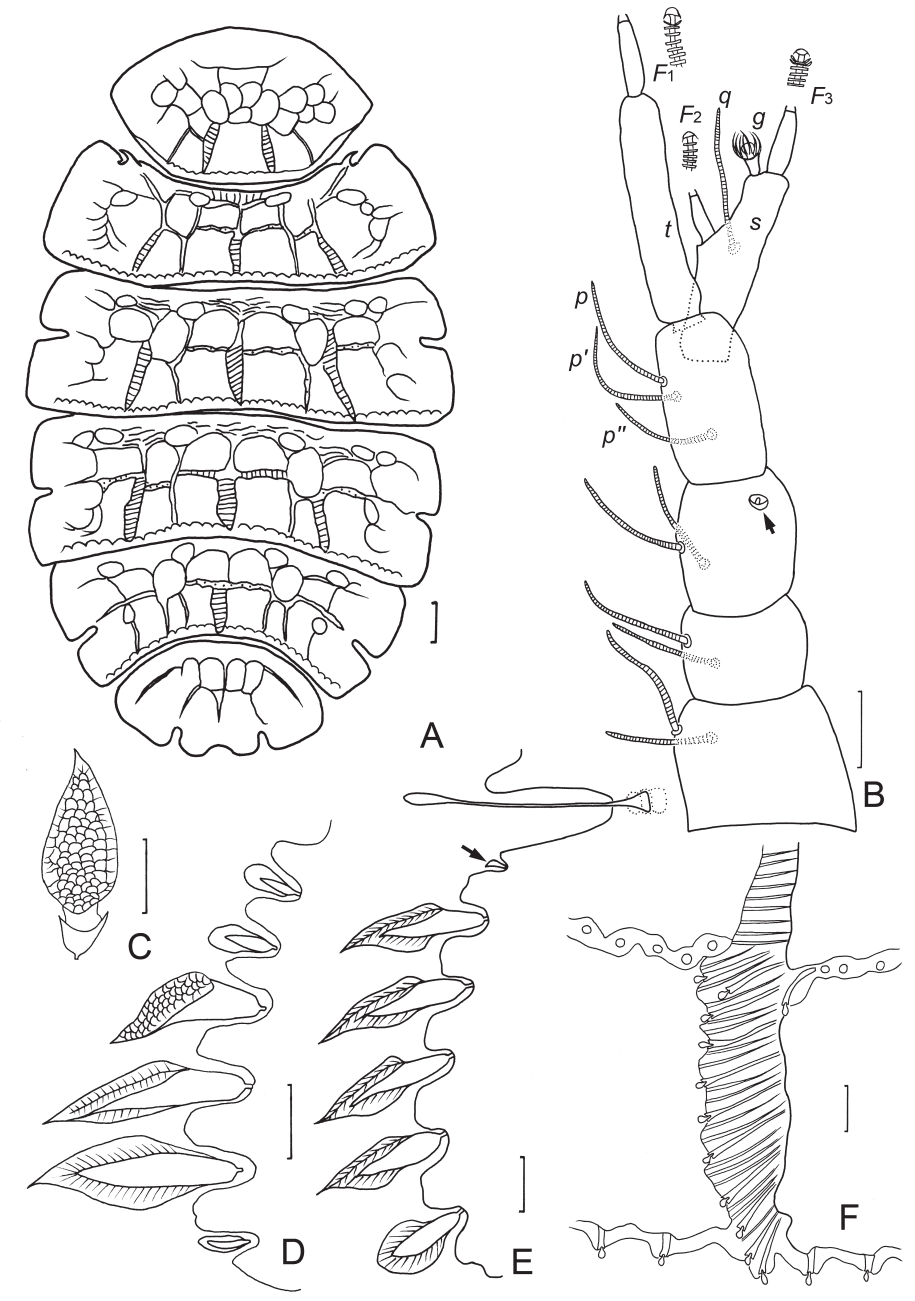
*Samarangopustestudineus* sp. nov. (holotype) **A** tergites I–VI, showing the testudinal pattern **B** right antenna, tergal view (arrow indicates pin-shaped seta) **C** marginal leaf-shaped protuberance, sternal view **D** left posterior corner of tergite I, tergal view **E** left posterior corner of tergite IV, sternal view (arrow indicates tiny rod-shaped protuberance) **F** ridges on the tergite IV, lateral view on slide. Scale bars: 100 μm (**A**); 20 μm (**B–F**).

Adult body length 1.95 mm; body dark brown in alcohol, brown to reddish after mounted on slides, dorsally with distinct testudinal pattern (Figs 1A, B, 2A).

***Head*** setae strongly reduced, dorsally with setae *a*_0_ and one pair of lateral setae *l*_1_, other setae absent. Temporal organs rectangular in tergal view, length 0.7 of shortest interdistance, glabrous. Tiny pistils present laterally.

***Antennae*** (Fig. 2B). Chaetotaxy of segments 1–4: 2/2/3/3. Setae thin, cylindrical, striate, length of setae on segment 4: *p* = 40 μm, *p*’ = 35 μm, *p*″ = 30 μm; *u* and *r* absent. Third antennal segment with two normal setae and one rudimentary pin-shaped seta. Tergal branch *t* cylindrical, 4.6 times as long as greatest diameter and 1.1 times as long as sternal branch *s*, the latter with distinct anterior indentation at level of *F*_2_, 3.2 times as long as greatest diameter. Seta *q* similar to setae of segment 4, 30 μm, 0.5 times of length of *s.* Globulus *g* with conical stalk, length of *g* (11 μm) 1.4 times as long as its greatest diameter; the latter 0.2 times of greatest diameter of *t*; 10 bracts, capsule spherical, diameter = 8 μm; stalk length 5 μm. Relative lengths of flagella (base segments included): *F*_1_ = 100, *F*_2_ = 55, *F*_3_ = 89. Lengths of base segments: *bs*_1_ = 20 μm, *bs*_2_ = 13 μm, *bs*_3_ = 18 μm. *F*_1_ 3.1 times as long as *t*, *F*_2_ and *F*_3_ 1.8 and 3.0 times as long as sternal branch *s*, respectively. Calyces of *F*_1_ largest, those of *F*_2_ and *F*_3_ smaller, all subhemispherical.

**Figure 3.**
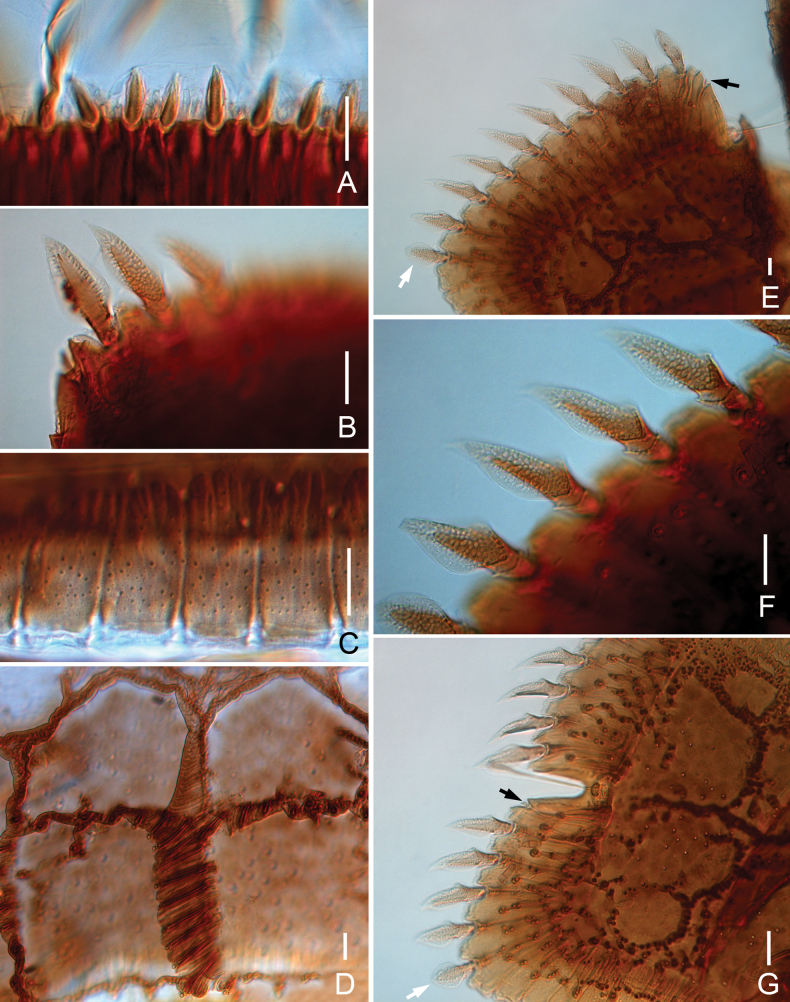
*Samarangopustestudineus* sp. nov. (holotype) **A** anterior margin of tergite I **B** left posterior corner of tergite I **C** hind margin of tergite I **D** ridges on tergite IV **E** left side of tergite II (black arrow indicates tiny rod-shaped protuberance, white arrow indicates round leaf-shaped) **F** marginal protuberances on tergite II, sternal view **G** tergite IV, left side (arrow indicates the same as in **E**). Scale bars: 20 μm.

**Figure 4.**
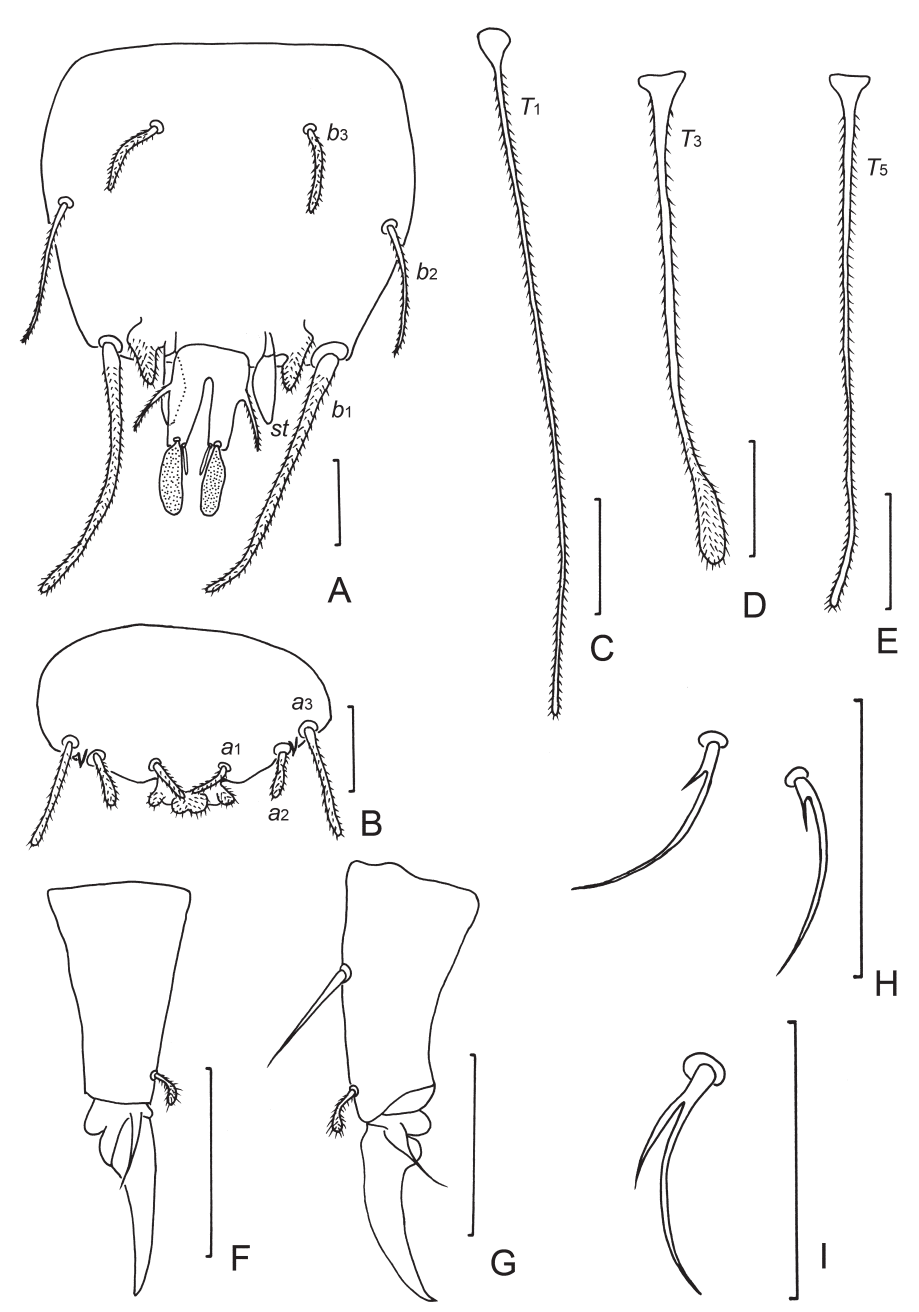
*Samarangopustestudineus* sp. nov. (holotype) **A** sternum of pygidum and anal plate **B** tergum of pygidum **C***T*_1_**D***T*_3_**E***T*_5_**F** tarsus of leg 1 **G** tarsus of leg 9 **H** setae on coxa and trochanter of leg 1 **I** setae on trochanter of leg 9. Scale bars: 20 μm.

***Trunk*.** Collum segment not clearly visible. Tergites with testudinal patterns limited by different kind of structures and protuberances (Figs 1A, B, 2A). Vertical wide ridges composed by long, candle-like protuberances located on tergites I–V medially, transverse narrow ridges composed by short, candle-like protuberances and conical protuberances located on tergites II–V (Figs 2F, 3D). Posterior margin of tergites comb-shaped with tiny granules on it (Fig. 3C). Cuticles between these structures coarse (Fig. 3D, G). Marginal protuberances well differentiated with different shapes: (1) conical on anterior margin and posterior corner of tergite I (Figs 2D, 3A); (2) pointed leaf-shaped with reticulations on posterolateral margin of tergite I and lateral margin of other tergites (Figs 2C–E, 3B, E, F, G); (3) one rounded leaf-shaped on the posterior corner of tergite II–V (Figs 2E, 3E, G); (4) tiny, rod-shaped on anterior corner of each tergite and behind cavities of bothriotricha of tergites II–V (Figs 2E, 3E, G). Pattern of marginal protuberances: tergite I: 1 tiny–3 large–41 small–3 large–1 tiny; tergite II: 1 small–1 tiny–*T*_1_–10 large; tergite III: 1 small–7 large–1 tiny–*T*_2_–6 large; tergite IV: 1 small–8 large–l tiny–*T*_3_–5 large; tergite V: 1 small–(8–10) large–1 tiny–*T*_4_–4 large; tergite VI: 1 small–(7–8)–*T*_5_–2 large. Length/width ratio of tergites: I = 0.58, II = 0.26, III = 0.30, IV = 0.29, V = 0.32, and VI = 0.58.

***Bothriotricha*.** All with short pubescence, *T*_1_, *T*_2_, *T*_4_, and *T*_5_ thin and with blunt apex (Fig. 4C, E), *T*_3_ shorter than others, with thicker axis, distal part spatulate, and densely pubescent (Fig. 4D). Relative lengths of bothriotricha: *T*_1_ = 100, *T*_2_ = 92, *T*_3_ = 75, *T*_4_ = 96, *T*_5_ = 81.

***Legs*.** All legs 5-segmented. Setae on coxa and trochanter of leg 9 similar to each other; thin, glabrous, bifurcate, with length of secondary branch 0.6 times of primary one (Fig. 4I). Tarsi tapering, those of leg 9 1.9 times as long as greatest diameter; proximal seta glabrous, pointed, 35 μm, 0.4 times of the length of tarsus (75 μm) and 2.9 times as long as distal pubescent seta (12 μm) (Fig. 4G). Tarsus of leg 1 with only pubescent distal seta (Fig. 4F). Setae on coxa and trochanter of leg 1 both bifurcate, glabrous, length of secondary branch 0.2 times of primary one (Fig. 4H). All legs with large main claw and small setose anterior secondary claw (Fig. 4F, G).

***Pygidium. Tergum*** (Fig. 4B). Setae pubescent: *a*_1_ and *a*_2_ short, clavate, the former curved inwards; *a*_3_ straight, cylindical. Three pubescent appendages of irregular shape: two lateral triangular appendages between *a*_2_ and *a*_3_, one medial appendage at posterior margin, located posterior to Setae *a*_1_. Lengths of setae: *a*_1_ = 12 μm, *a*_2_ = 13 μm, *a*_3_ = 28 μm. Distances *a*_1_–*a*_1_ = 13 μm, *a*_1_–*a*_2_ = 12 μm, *a*_2_–*a*_3_ = 4 μm.

***Sternum*** (Fig. 4A). Setae pubescent: *b*_1_ and *b*_3_ thick, with blunt apex. Seta *b*_2_ slender, pointed, tapering. Lengths of setae: *b*_1_ = 70 μm, *b*_2_ = 33 μm, *b*_3_ = 22 μm. Distance *b*_1_–*b*_1_ = 48 μm, *b*_2_–*b*_2_ = 78 μm, *b*_1_–*b*_2_ = 30 μm, *b*_3_–*b*_3_ = 36 μm. Seta *b*_1_ 1.5 times as long as interdistance, *b*_2_ 1.1 times as long as distance *b*_1_–*b*_2_, *b*_3_ 0.6 times of interdistance. *st* leaf-shaped, glabrous, 18 μm in length, *st*–*st* = 20 μm (Fig. 4B). Posterior margin between *b*_1_ straight. Two pubescent, triangular appendages present between *b*_1_ and anal plate.

***Anal plate*** (Fig. 4A) 1.8 times as long as broad, slightly tapering posteriorly; lateral margins with one pair of thin, diverging, pubescent branches, 0.5 times of the length of plate; posterior 2/3 of plate divided into two tapering branches by a deep, V-shaped incision, each branch with two apical appendages: a submedian short, straight, tapering, glabrous one and a stalked bladder, sausage-shaped in sternal view. Bladder 0.7 times as long as plate. Plate glabrous, bladder densely granulated.

##### Etymology.

From the masculine Latin word “*testudineus*” meaning “with the pattern of tortoise shell” that refers to the testudinal pattern on the tergites of the new species.

##### Distribution.

China (Hunan). Known only from the type locality.

##### Remarks.

*Samarangopustestudineus* sp. nov. can be easily distinguished from all other congeners by the unique dorsal testudinal pattern on the body and the shape of protuberances on the body, as well as the anal plate. The dark-brown ridges composed of different structures and protuberances on tergites were only observed in *S.amplissimus* Scheller, 2009 from Indonesia, but their patterns are apparently different between the two species (vertically located on posterior part of tergites I–V in *S.testudineus* sp. nov. vs located on anterior part of tergite I and lateral part of tergites II–VI, curved). The species also differ in the shapes of marginal protuberances on tergite I (differentiated in three kinds, with pattern 1 tiny–3 large–41 small–3 large–1 tiny in *S.testudineus* sp. nov. vs with 38 similar leaf-shaped, large protuberances in *S.amplissimus*), the shape of leaf-shaped protuberances (with reticulations in *S.testudineus* sp. nov. vs without reticulations in *S.amplissimus*), the shape of globulus *g* on antenna (1.4 times as long as greatest diameter in *S.testudineus* sp. nov. vs 2.4 times as long as greatest diameter in *S.amplissimus*), the shape of the setae on the pygidial sternum (cylindrical in *S.testudineus* sp. nov. vs slender and pointed in *S.amplissimus*), and the anal plate (with sausage-shaped, granulated bladders in *S.testudineus* sp. nov. vs with ovoid, pubescent bladders in *S.amplissimus*).

#### ﻿ Samarangopus rotundifolius sp. nov.

Taxon classificationAnimaliaTetramerocerataEurypauropodidae

B7EA8C0E-9D5F-5807-B96C-EC558197E623

https://zoobank.org/730405EE-7555-4FEE-B9F9-E08AF42F1450

Figs 5 , 6 , 7

##### Material examined.

***Holotype***, male adult with 9 pairs of legs (slide no. ZJ-GTS-PA2012011) (**SNHM**), China, Zhejiang Province, Gutian Mountain, extracted from soil samples in the broad-leaved forest, Alt. 1000 m, 29°16'N, 118°06'E, 11-IV-2012, coll. Y. Bu. ***Paratype***, 1 male adult with 9 pairs of legs (slide no. ZJ-GTS-PA2012012), same data as holotype. **Non-type specimens**, 2 juveniles with 6 pairs of legs (slides no. ZJ-GTS-PA2012028, ZJ-GTS-PA2012029), 1 juvenile with 5 pairs of legs (slide no. ZJ-GTS-PA2012030), same data as holotype.

##### Diagnosis.

*Samarangopusrotundifolius* sp. nov. is characterized by large, round, leaf-shaped protuberances on the anterior margin of tergite I and the lateral margins of tergites I–VI, small, candle-like protuberances with distal, flame-like structures and entire protuberance surrounded by a circular collar mainly situated in the caudal halves of all tergites, trifurcated setae on coxa and trochanter of leg 1, and a pair of triangular bladders on the anal plate.

##### Description.

**Figure 5.**
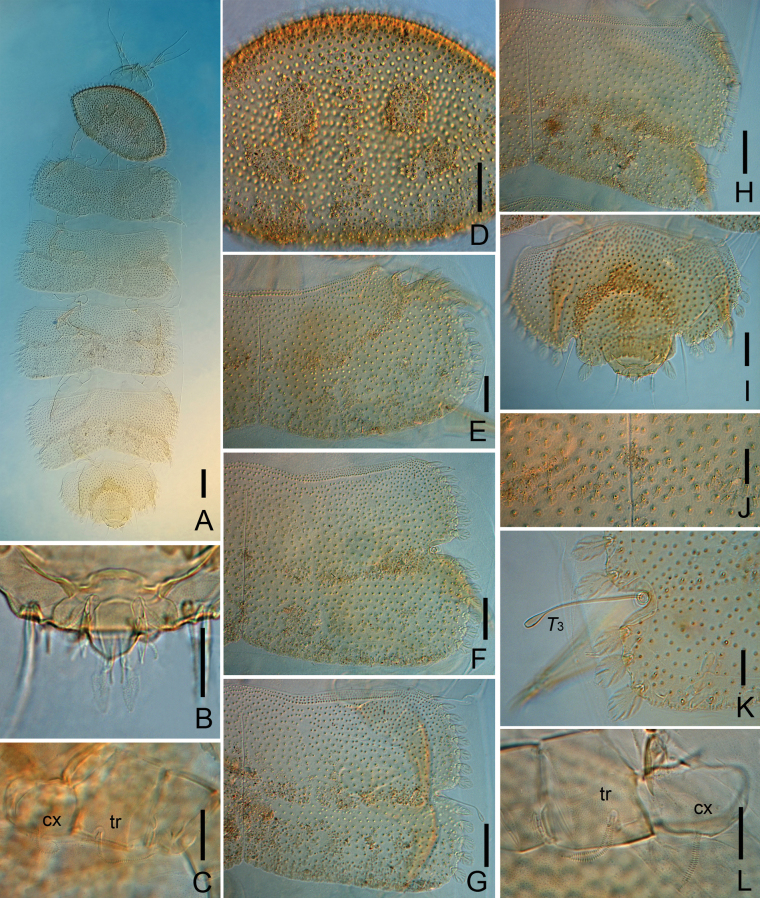
*Samarangopusrotundifolius* sp. nov. (holotype) **A** habitus, tergal view, on slide **B** anal plate **C** setae on coxa (cx) and trochanter (tr) of leg 1 **D** tergite I **E** tergite II, right side **F** tergite III, right side **G** tergite IV, right side **H** tergite V right side **I** tergite VI and pygidum **J** middle part of tergite II, showing protuberances **K** left side of tergite IV, with round, leaf-shaped protuberance and *T*_3_**L** setae on coxa (cx) and trochanter (tr) of leg 9. Scale bars: 100 μm (**A**); 20 μm (**B–L**).

Adult body length (1.4–) 1.5 mm (*n* = 2); body brown to yellow (Fig. 5A).

**Figure 6.**
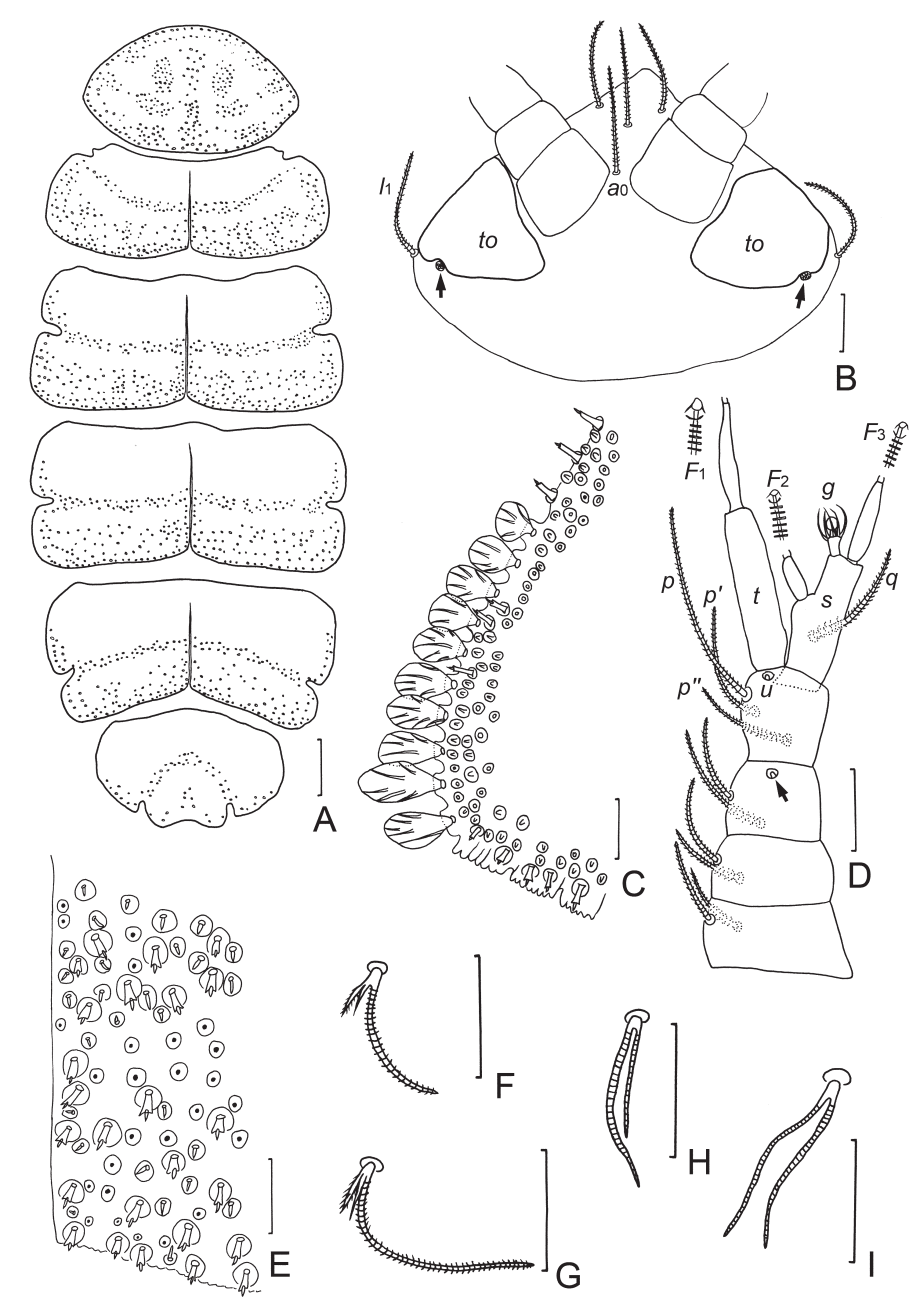
*Samarangopusrotundifolius* sp. nov. (holotype) **A** tergites I–VI, showing the pattern of candle-shaped protuberances on the body **B** head, tergal view (*to*–temporal organ; arrows indicate pistils) **C** left posterior corner of tergite I **D** right antenna, tergal view (arrow indicates pin-shaped seta) **E** middle part of tergite II, with candle-like and conical protuberances **F** seta on coxa of leg 1 **G** seta on trochanter of leg 1 **H** seta on the coxa of leg 9 **I** seta on the trochanter of leg 9. Scale bars: 100 μm (**A**); 20 μm (**B–I**).

***Head*** (Fig. 6B) setae strongly reduced, with setae *a*_0_ (33 μm) on dorsal surface and one pair of lateral setae *l*_1_ (35 μm), other dorsal setae absent. Temporal organs rectangular in tergal view, length 0.8 of shortest interdistance, glabrous. Tiny pistils present laterally.

***Antennae*** (Fig. 6D). Chaetotaxy of segments 1–4: 2/2/3/4. Setae thin, tapering, striate, length of setae on segment 4: *p* = 45 μm, *p*’ = 28 (–30) μm, *p*″ = 28 (–30) μm, *u* present and *r* absent. Third antennal segment with two normal setae and one rudimentary, pin-shaped setae. Tergal branch *t* cylindrical, 4.0 (–4.7) times as long as greatest diameter and 1.2 times as long as sternal branch *s*; the latter with distinct anterior indentation at level of *F*_2_, 2.6 (–2.9) times as long as greatest diameter. Seta *q* similar to setae of segment 4, (25–) 30 μm, (0.7–) 0.9 times of the length of *s.* Globulus *g* with conical stalk, length of *g* (10 μm) (1.0–) 1.2 times as long as its greatest diameter; the latter 0.2 times of greatest diameter of *t*; 12 bracts, capsule spherical, diameter = 8 μm; stalk length 10 μm. Relative lengths of flagella (base segments included): *F*_1_ = 100, *F*_2_ = 46 (–48), *F*_3_ = 84 (–92). Lengths of base segments: *bs*_1_ = (26–) 28 μm, *bs*_2_ = 10 μm, *bs*_3_ = (18–) 20 μm. *F*_1_ (2.9–) 3.1 times as long as *t*, *F*_2_ and *F*_3_ 1.7 and 3.1 (–3.3) times as long as sternal branch *s*, respectively. Calyces of *F*_1_ largest, those of *F*_2_ and *F*_3_ smaller, all subhemispherical.

**Figure 7.**
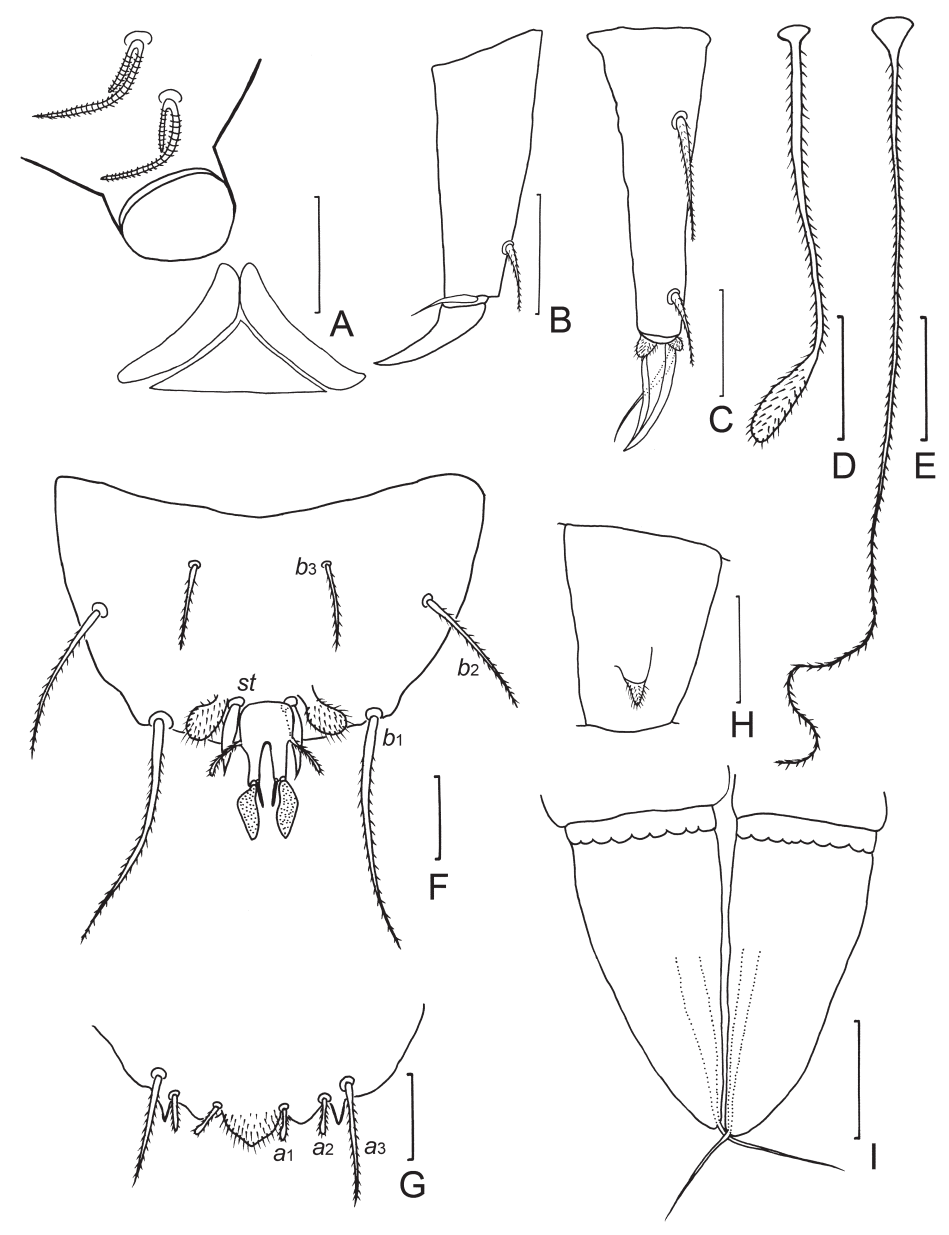
*Samarangopusrotundifolius* sp. nov. (holotype) **A** collum segment, sternal view **B** tarsus of leg 1 **C** tarsus of leg 9 **D***T*_3_**E***T*_5_**F** sternum of pygidium and anal plate **G** tergum of pygidium **H** femur of leg 1 with appendage **I** male genital papillae. Scale bars: 20 μm.

***Trunk*.** Setae of collum segment uniform, furcate, branches cylindrical and striate; both setae length 20 μm (Fig. 7A). Appendages barrel-shaped; caps flat (Fig. 7A). Sternite process broad, with anterior V-shaped incision. Tergites densely covered with protuberances of different shapes (Figs 5A, D–K, 6A, C, E). Tergites II–V incompletely 2-partitioned posteriorly by a narrow, median, longitudinal groove; tergites I and VI entire (Fig. 6A). Three main types of protuberances: large and round, leaf-shaped protuberances present on anterior margin of tergite I and lateral margins of tergites I–VI (Figs 5E–I, K, 6C); small, candle-like protuberances each surrounded by a circular collar (Fig. 6C, E); tiny, conical protuberances with circular collar (Fig. 6C, E). Distribution pattern of candle-like protuberances as shown in Fig. 6A. Cuticle between these structures glabrous (Fig. 5D–K). Anterior margin of tergites II–VI with 3–5 rows of regular coarse granules (Fig. 5E–I). Pattern of marginal protuberances: tergite I: 40; tergite II: 1 small–*T*_1_–10; tergite III: 1 small–7–*T*_2_–7; tergite IV: 1 small–7 (8–9)–*T*_3_–5; tergite V: 9–*T*_4_–4 (3–4); tergite VI; 7 (8)–*T*_5_–1. Length/width ratio of tergites: I = 0.59(–0.63), II = 0.38(–0.4), III = (0.45–)0.48, IV = (0.45–)0.48, V = 0.48, and VI = 0.59(–0.67).

***Bothriotricha*.** All with short pubescence, *T*_1_, *T*_2_, *T*_4_, and *T*_5_ thin and with distal part curled (Fig. 7E), *T*_3_ shorter than others, with thicker axis, distal part spatulate and densely pubescent (Figs 5K, 7D). Relative lengths of bothriotricha: *T*_1_ = 100, *T*_2_ = 120(–125), *T*_3_ = 38(–45), *T*_4_ = 60(–68), *T*_5_ = 75(–77).

***Legs*.** All legs 5-segmented. Setae on coxa and trochanter of leg 1 both trifurcated, striate, two short branches 0.2 times of primary one, with middle one glabrous and lateral one pubescent (Figs 5C, 6F, G). Tarsus of leg 1 with a single pubescent distal seta (Fig. 7B). All legs with large main claw and small setose anterior secondary claw (Figs 7B, C). Setae on coxa and trochanter of leg 9 thin (Figs 5L, 6H, I), furcate, and striate, length of secondary branch 0.9 times of primary one on trochanter (Fig. 6H), subequal on coxa (Fig. 6I). Tarsi tapering, those of leg 9 (2.6–)3.0 times as long as greatest diameter; setae pubescent, tapering, pointed, proximal one (25–)30 μm, 0.4 times of the length of tarsus (65 μm) and 2.1(–2.5) times as long as distal one (12 μm) (Fig. 7C). Anterior side of femur of leg 1 with a single conical pubescent plate (Fig. 7H).

***Male genital papillae*** (Fig. 7I). Base segments cylindrical. Length of papillae = 75 μm, greatest diameter = 32 μm, length of seta = 25 μm. Proximal part of genital papillae subcylindrical, distal part conical, seta 0.3 times of length of papilla. Cuticle glabrous.

***Pygidium. Tergum*** (Fig. 7G). Setae pubescent: *a*_1_ and *a*_2_ short and clavate; *a*_3_ straight, pointed. A median, unpaired linguiform, pubescent appendage framed by the paired seta *a*_1_. Posterior margin with a pair of lateral triangular appendages situated between stae *a*_2_ and *a*_3_ of each side. Lengths of setae: *a*_1_ = *a*_2_ = 10 μm, *a*_3_ = (24–)26 μm. Distances *a*_1_–*a*_1_ = 14 μm, *a*_1_–*a*_2_ = 8 μm, *a*_2_–*a*_3_ = 5 μm.

***Sternum*** (Fig. 7F). Setae pubescent: *b*_2_ and *b*_3_ thin, pointed. Seta *b*_1_ thick, long, tapering, pointed. Lengths of setae: *b*_1_ = 56(–60) μm, *b*_2_ = 33 μm, *b*_3_ = 20 μm. Distance *b*_1_–*b*_1_ = 50 μm, *b*_2_–*b*_2_ = 78 μm, *b*_1_–*b*_2_ = 26 μm, *b*_3_–*b*_3_ = 30 μm. Seta *b*_1_ 1.2 times as long as interdistance, *b*_2_ 1.3 times as long as distance *b*_1_–*b*_2_, *b*_3_ 0.7 times of interdistance. Posterior margin of sternum between *b*_1_ slightly rounded. Between *b*_1_ and anal plate, a pair of pubescent oval appendages present and a pair of lanceolate, glabrous styli *st. st* = 18 μm, *st*–*st* = 13 μm.

***Anal plate*** (Figs 5B, 7F) 1.6 times as long as broad, slightly tapering posteriorly; lateral margins with a pair of thin, diverging, pubescent branches, 0.5 times of the length of plate, distal part faintly inflated; posterior 1/2 of plate divided into two tapering branches by a deep, V-shaped incision, each branch with two apical appendages: a submedian short, straight, glabrous one and a stalked bladder of triangular shape in sternal view. Bladder 0.6 times of length of plate. Plate glabrous, bladder densely granulated.

##### Etymology.

From the Latin “*rotundus*” = “round” and “*folium*” = “of leaf”. The species name “*rotundifolius*” is masculine that refers to the round, leaf-shaped protuberances on the margin of tergites in the new species.

##### Distribution.

China (Zhejiang). Known only from the type locality.

##### Remarks.

*Samarangopusrotundifolius* sp. nov. can be easily distinguished from all other congeners by the round, leaf-shaped marginal protuberances on its tergites. It is similar to *S.umbonifer* Scheller, 1995 and *S.doiinthanonaeus* Scheller, 1995 from Thailand in the shape of the anal plate and setae on the pygidium. They differ in the shape of marginal protuberances on tergite I (all rounded leaf-shaped in *S.rotundifolius* sp. nov. vs fungiform at anterior and anterolateral margins and some wedge- to leaf-shaped at posterolateral corners in *S.umbonifer*, and all wedge- to leaf-shaped in *S.doiinthanonaeus*), the shape of setae on the collum segment (furcate and the secondary branch about half length of primary one in *S.rotundifolius* sp. nov. vs furcate with a rudimentary secondary branch in *S.umbonifer* and *S.doiinthanonaeus*), shape of setae on tergum of pygidium (*a*_1_ and *a*_2_ short, clavate, pubescent, subequal in *S.rotundifolius* sp. nov. vs *a*_1_ cylindrical and longer than clavate *a*_2_, both glabrous in *S.umbonifer*, and *a*_1_ and *a*_2_ both cylindrical, pubescent in *S.doiinthanonaeus*), and the shape of the plate on the anterior side of the femur of leg 1 (conical in *S.rotundifolius* sp. nov. vs linguiform and slightly pointed in *S.umbonifer*, linguiform and round in *S.doiinthanonaeus*).

#### ﻿Genus Eurypauropus

Taxon classificationAnimaliaTetramerocerataEurypauropodidae

Ryder, 1879

60AB7F11-6F21-57E9-A41C-2A2910DF890B

##### Type species.

*Eurypauropusspinosus* Ryder, 1879.

##### Diagnosis.

Fourth antennal segment with four well-developed setae; globulus *g* of ventral antennal branch long-stalked; third antennal segment with a globulus *g*_2_; setae of tergites inserted in rounded crater-shaped structures; first and last pair of legs 5-segmented, other pairs 6-segmented; anal plate V-shaped with straight lateral margins; interdistance of pygidial setae *a*_1_ nearly twice as long as distance *a*_2_–*a*_3_ (Scheller 2011).

##### Distribution.

Nearctic, Palaearctic.

#### ﻿ Eurypauropus japonicus

Taxon classificationAnimaliaTetramerocerataEurypauropodidae

Hagino & Scheller, 1985, new record to China

87B8D062-DA13-5A29-8B7E-9069F6C0745D

Fig. 8

##### Material examined.

1 female adult with 9 pairs of legs (slide no. ZJ-GTS-PA2012023), China, Zhejiang Province, Gutian Mountain, extracted from soil samples in broad-leaved forest, alt. 1000 m, 29°16'N, 118°06'E, 27-III-2013, coll. Y. Bu.

##### Diagnosis.

*Eurypauropusjaponicus* is characterized by the shape of the anal plate with one pair of small, pointed lateral appendages, subcylindrical setae *b*_2_ on the sternum of the pygidium, tergites with large, curved, ciliated spines and small, nipple-shaped tubercles with conical bases.

##### Description of new material.

**Figure 8.**
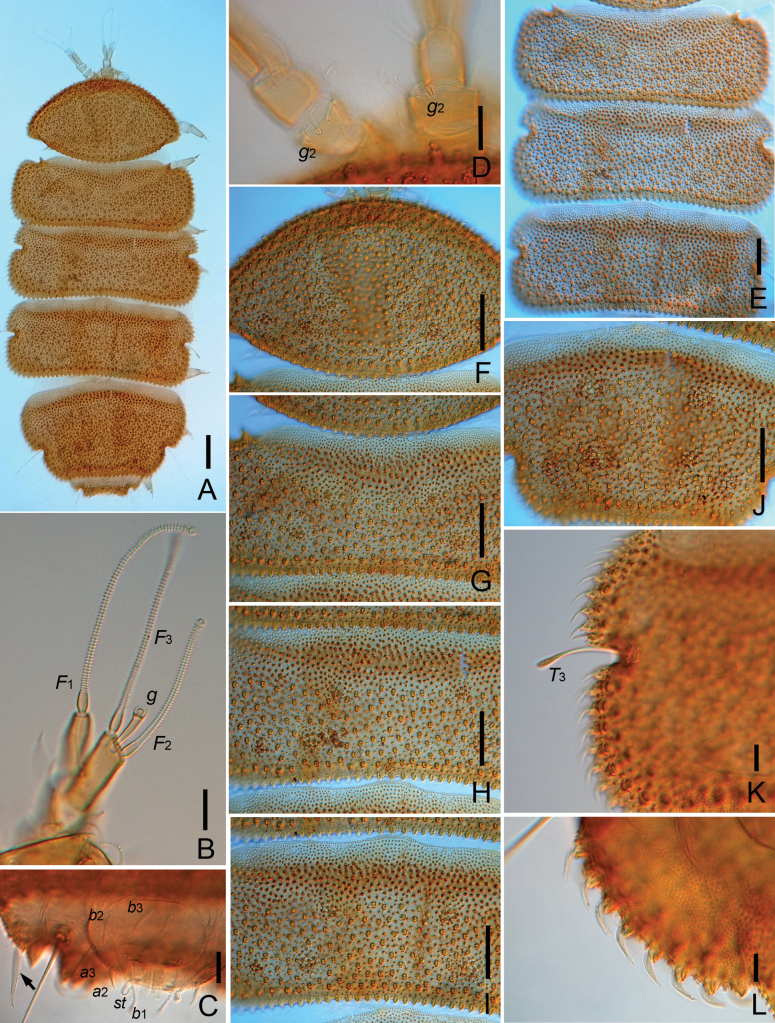
*Eurypauropusjaponicus* Hagino & Scheller, 1985 (Chinese specimen) **A** habitus, tergal view, on slide **B** right antenna, tergal view **C** posterior corner of tergite VI, pygidium and anal plate (arrow indicates the big spine), sternal view **D** antennal segment III, showing globulus *g*_2_**E** Tergite II–IV **F** tergites I **G** tergite II, middle part **H** tergite III, middle part **I** tergite IV, middle part **J** tergite V **K** tergite IV, left side, showing the marginal protuberances and *T*_3_**L** tergite V, left side, showing marginal protuberances. Scale bars: 100 μm (**A**); 20 μm (**B–L**).

Length 1.28 mm, light brown (Fig. 8A). Head covered by tergite I and chaetotaxy not observed in detail.

***Antennae*** (Fig. 8B, D). Chaetotaxy of segments 1–4: 2/2/4/5. Setae cylindrical, annulate. Length of setae on segment 4: *p* = 40 μm, *p*’ = 26 μm, *p*″ = 25 μm; *p*‴ = 21; *r* = 15 μm, *u* absent. Tergal branch *t* fusiform, 3.3 times as wide as greatest diameter and 0.8 times as long as sternal branch. Sternal branch *s* with distinct anterior indentation at level of *F*_2_, 2.6 times as long as greatest diameter. Seta *q* similar to setae of segment IV, 40 μm, 0.8 times the length of *s.* Globulus *g* with long, cylindrical stalk, length of *g* (27 μm) 3.8 times as long as greatest diameter; the latter 0.2 times of greatest diameter of *t*; 10 bracts, capsule spherical, diameter = 5 μm; stalk length 20 μm. Globulus *g*_2_ on third antennal segment with short, pubescent stalk, 6 μm in length, 2.2 times as long as greatest diameter, capsule tiny, diameter = 2.5 μm, stalk length 4 μm. Relative lengths of flagella (base segments included): *F*_1_ = 100, *F*_2_ = 69, *F*_3_ = 88. Lengths of base segments: *bs*_1_ = 12 μm, *bs*_2_ = 11 μm, *bs*_3_ = 13 μm. *F*_1_ 3.3 times as long as *t*, *F*_2_ and *F*_3_ 1.9 and 2.4 times as long as sternal branch *s*, respectively. Calyces of *F*_1_ largest, conical, those of *F*_2_ and *F*_3_ smaller, subhemispherical.

***Trunk*.** Setae of collum segment not clearly seen. Tergites densely covered with two types of protuberances: large, curved, evenly distributed, spiniform protuberances and small, nipple-shaped tubercles with conical bases (Fig. 8 F–L). The former distinct and long on marginal parts of tergites (Fig. 8K, L) but absent on anterior parts of tergites II–VI (Fig. 8G–J). Tergite I–V each with six open fields without protuberances but with circular tubercles of medium size. Posterior margin of tergites II–V with one regular row of protuberances (Fig. 8A, E, G–J). One large spine (40 μm) present on the posterior corner of tergite VI (Fig. 8C). Pattern of marginal protuberances: tergite I: 40; tergite II: *T*_1_–19; tergite III: 7–*T*_2_–l2; tergite IV: 8–*T*_3_–l0; tergite V: (8–10)–*T*_4_–(6–8); tergite VI: 1 spine–*T*_5_–2. Length/width ratio of tergites: I = 0.62, II = 0.35, III = 0.39, IV = 0.41, V = 0.56, VI = 0.32.

***Bothriotricha*.***T*_1_ and *T*_2_ with thin axes and glabrous proximal parts, medial part with erect, short pubescence, and distal 4/5 with branched hairs arranged in whorls. *T*_3_ shorter than others, club-like, and glabrous (Fig. 8K). *T*_4_ and *T*_5_ with thin axes and glabrous. Relative lengths of bothriotricha: *T*_1_ = 100, *T*_2_ = 107, *T*_3_ = 50, *T*_4_ = 93, *T*_5_ = 83.

***Legs*.** Legs 1 and 9 both 5-segmented, others 6-segmented. Setae on coxa and trochanter of leg 9 similar to each other, bifurcate, densely annulated, length of secondary branch subequal to primary one. Tarsus of leg 9 thick, tapering, 1.6 times as long as greatest diameter; 2 tergal setae and 1 sternal setae pointed, glabrous; proximal seta length 23 μm, 0.4 times of the length of tarsus (52 μm) and 1.9 times as long as distal seta (13 μm). Main claw 27 μm, 0.5 times as long as tarsus, anterior accessory claw tapering (17 μm). Cuticle of tarsus with minute granules. Tarsus of leg 1 with 1 tergal seta (13 μm) and 1 sternal seta (15 μm), both glabrous and pointed, main claw 27 μm and accessory claw 10 μm.

***Pygidium. Tergum.*** Posterior margin round. Seta *a*_1_ short, cylindrical, pubescent; *a*_2_ and *a*_3_ spiniform, glabrous; *a*_3_ sharply pointed (Fig. 8C). Lengths of setae: *a*_1_ = 13 μm, *a*_2_ = 17 μm, *a*_3_ = 36 μm. Distance *a*_1_–*a*_1_ = 23 μm, *a*_1_–*a*_2_ = 15 μm, *a*_2_–*a*_3_ = 12 μm.

***Sternum*** (Fig. 8C). Posterior margin between *b*_1_ with two low, median, rounded lobes. All setae cylindrical, blunt, and pubescent, *b*_1_ with broad base and distal weak swelling, *b*_2_ and *b*_3_ short. Lengths of setae: *b*_1_ = 40 μm, *b*_2_ = 20 μm, *b*_3_ = 20 μm. Distances *b*_1_–*b*_1_ = 32 μm, *b*_2_–*b*_2_ = 60 μm, *b*_1_–*b*_2_ = 28 μm, *b*_3_–*b*_3_ = 10 μm. *b*_1_ 1.2 times as long as interdistance, *b*_2_ 0.7 of distance *b*_1_ –*b*_2_, *b*_3_ 2.0 of interdistance. Styli *st* slender, cylindrical, pubescent, and curved, 20 μm, *st*–*st* = 30 μm.

***Anal plate*.** 1.1 times as long as broad; narrow at base; distal part of plate cleft by narrow, V-shaped incision, depth about half of plate length, incision forming two posterior branches, each carrying two pairs of appendages: submedian pair leaf-shaped, about half length of plate, 2.1 times as long and wide; lateral ones short, pointed and pubescent. Plate glabrous, distal appendages pubescent (Fig. 8C).

##### Distribution.

China (Zhejiang), Japan (Honshu, Kyushu).

##### Remarks.

*Eurypauropusjaponicus* was originally described and known from Honshu and Kyushu, Japan (Hagino and Scheller 1985; Hagino 1992). The antenna, protuberances, and bothriotricha on tergites, the setae on legs and pygidium, and the shape of the anal plate of Chinese specimens are very similar to *E.japonicus*, which corroborates the species identity. The main difference observed are: (1) the protuberances on the lateral margin of tergites which are thin and pointed (thick and blunt in types); (2) tergites I–V each with 6 open fields have circular tubercles (eight in types); (3) the bothriotricha *T*_1_ and *T*_2_ are medially with erect, short pubescence and the distal 4/5 have distinct, branched hairs arranged in whorls (distal 1/3 with short pubescence in types). Other minor differences are body size, lengths of setae, bothriotricha, and flagella, which might be due to the variation between populations. In addition, one pair of large spines located on the posterior corner of tergite VI observed in Chinese specimens was not mentioned in the original description of type materials.

## ﻿Discussion

The genus *Samarangopus* includes 40 species distributed in the Palearctic, Ethiopian, and Oriental regions (Scheller 2011; Qian et al 2014; Bu 2020c). The genus is rich in species in South and East Asia, with 28 species recorded: five species in China, four in Thailand, two in Philippines, seven each in Malaysia and Indonesia, and one each in Vietnam, Singapore, and Nepal. The remained 12 species are described from New Caledonia (four species), Madagascar (three), New Zealand (two), Papua New Guinea (one), Australia (one), and Rwanda (one). They usually living in upper layer of soil or in litter of forests, but with lower density compared with pauropods of the family Pauropodidae. They can be easily separated from other groups of pauropods by the strongly sclerotized tergites and relatively flattened body.

The genus *Eurypauropos* includes about 10 species recorded from USA and Japan (Scheller 2011; https://millibase.org/). It is a group belong to Nearctic and Palaearctic regions and also have strong sclerotized tergites and a robust habitus. In China, only one undetermined species, *Eurypauropus* sp., was ever reported from Zhejiang Province (Zhang and Chen 1988). The present paper determines the first species of the genus from China, *E.japonicus*, which was only found in Japan before.

## Supplementary Material

XML Treatment for Samarangopus

XML Treatment for Samarangopus testudineus

XML Treatment for Samarangopus rotundifolius

XML Treatment for Eurypauropus

XML Treatment for Eurypauropus japonicus

## ﻿Additional information

### Author ORCIDs

Yun Bu https://orcid.org/0000-0002-7177-9686

### Data availability

All of the data that support the findings of this study are available in the main text or Supplementary Information.
